# Cotton-Type and Joint Invariants for Linear Elliptic Systems

**DOI:** 10.1155/2013/540705

**Published:** 2013-12-25

**Authors:** A. Aslam, F. M. Mahomed

**Affiliations:** ^1^Differential Equations, Continuum Mechanics and Applications, School of Computational and Applied Mathematics, University of the Witwatersrand, Wits 2050, South Africa; ^2^School of Natural Sciences (SNS), National University of Sciences and Technology, Campus H-12, Islamabad 44000, Pakistan

## Abstract

Cotton-type invariants for a subclass of a system of two linear elliptic equations, obtainable from a complex base linear elliptic equation, are derived both by spliting of the corresponding complex Cotton invariants of the base complex equation and from the Laplace-type invariants of the system of linear hyperbolic equations equivalent to the system of linear elliptic equations via linear complex transformations of the independent variables. It is shown that Cotton-type invariants derived from these two approaches are identical. Furthermore, Cotton-type and joint invariants for a general system of two linear elliptic equations are also obtained from the Laplace-type and joint invariants for a system of two linear hyperbolic equations equivalent to the system of linear elliptic equations by complex changes of the independent variables. Examples are presented to illustrate the results.

## 1. Introduction

The general scalar linear second order partial differential equation (PDE) in two independent variables (*x*, *y*) is of the form

(1) $$\begin{array}{c} aw_{xx}+2bw_{xy}+cw_{yy}+dw_{x}+ew_{y}+fw=g, \end{array}$$

where *a*, *b*, *c*, *d*, *e*, *f*, and *g* are given *C* ^2^ functions of *x* and *y*. All linear PDEs of the form (1) are hyperbolic, elliptic, or parabolic depending on whether *b* ^2^ − *ac* is positive, negative, or zero, respectively, and thus can be simplified to one of the three canonical forms by introducing new coordinates [1]. The general linear second-order PDE (1) was first classified by Lie [2] in terms of its symmetry properties. He obtained seven canonical forms according to their point symmetries and types of equations. Of these, four belonged to the hyperbolic class and three to the parabolic class.

The scalar linear second order elliptic equation in two independent variables in canonical form is

(2) $$\begin{array}{c} u_{xx}+u_{yy}+au_{x}+bu_{y}+cu=0. \end{array}$$

It is well known that by means of the linear complex transformations [1, 3]

(3) $$\begin{array}{c} x=\frac{1}{2}\left(t+z\right),\;\;y=\frac{-i}{2}\left(t-z\right), \end{array}$$

the elliptic equation (2) can be mapped to the linear hyperbolic equation

(4) $$\begin{array}{c} u_{tz}+Au_{t}+Bu_{z}+Cu=0, \end{array}$$

where

(5) $$\begin{array}{c} A=\frac{1}{4}\left(a+ib\right),\;\;B=\frac{1}{4}\left(a-ib\right),\;\;C=\frac{1}{4}c. \end{array}$$

In 1773, Laplace [4] in his fundamental memoir deduced two semi-invariants

(6) h=At+AB−C,k=BZ+AB−C,

for (4), known as the Laplace invariants.

These Laplace invariants (6) can be transformed, by use of the inverse of the transformations (3) as well as after the substitution of (5) into (6) and then splitting the real and imaginary parts, to arrive at the Cotton invariants

(7) $$\begin{array}{c} \mu =a_{y}-b_{x},\\ H=a_{x}+b_{y}+\frac{1}{2}\left(a^{2}+b^{2}\right)-2c. \end{array}$$

These invariants (7) were first derived by Cotton [5]. Laplace and Cotton invariants remain unaltered under linear transformations of the dependent variable which, respectively, map the linear hyperbolic and elliptic equations into themselves. The corresponding invariant quantities for the linear parabolic equations can be found in [6–8]. Ovsiannikov [9] used the Laplace invariants in the group classification of the hyperbolic equation (4) by writing the determining equations for the symmetries of (4) in terms of these invariants. The solution of the equivalence problem for scalar linear (1 + 1) hyperbolic equations and some new invariants are given in [10, 11]. Laplace-type and joint invariants for a system of two linear hyperbolic equations are derived in [12] and Laplace-type invariants for a subclass of a system of two linear hyperbolic equations obtained from a complex linear hyperbolic equation are presented in [13]. The approach of complex symmetry analysis (CSA), was utilized in [14]. This method provides a connection between a complex scalar ordinary differential equation (ODE)/PDE and a system of real ODEs/PDEs by a complex split of the base complex equation into real and imaginary parts. In this work, we derive Cotton-type invariants for a subclass of a system of two linear hyperbolic PDEs. Cotton-type and joint invariants for a general linear system of elliptic equations are also determined. Examples are provided as illustration.

The outline of this note is as follows. In Section 2, Cotton-type invariants are derived for a subsystem of two linear elliptic equations by split of the complex Cotton invariants for the corresponding scalar complex linear elliptic equation. Cotton-type invariants for the same class are also obtained by transforming the Laplace-type invariants for the corresponding system of two linear hyperbolic equations which are equivalent to the system of two linear elliptic equations by means of complex linear transformations of the independent variables. These are shown to be the same. Moreover, Cotton-type and joint invariants for a general linear system of two elliptic equations are derived in Section 3. Then in Section 4 some examples are given to illustrate the results. Finally, in Section 5, a brief conclusion is given.

## 2. Cotton Invariants for a Subclass

In this section, Cotton-type invariants for a subsystem of two linear elliptic equations are first obtained from a complex scalar linear elliptic equation by splitting the complex Cotton invariants of the base complex equation into real and imaginary parts. Then for such a system, we determine invariants from the Laplace-type invariants for the equivalent system of two linear hyperbolic equations. This is achieved by performing complex splits of the Laplace-type invariants. It is concluded, as a proposition, that the Cotton-type invariants are the same for both the approaches. The subsystem of two elliptic equations

(8) $$\begin{array}{c} u_{xx}+u_{yy}+\alpha_{1}u_{x}-\alpha_{2}v_{x}+\beta_{1}u_{y}-\beta_{2}v_{y}+\gamma_{1}u-\gamma_{2}v=0,\\ v_{xx}+v_{yy}+\alpha_{2}u_{x}+\alpha_{1}v_{x}+\beta_{2}u_{y}+\beta_{1}v_{y}+\gamma_{2}u+\gamma_{1}v=0 \end{array}$$

is obtained by splitting of the complex linear elliptic equation

(9) $$\begin{array}{c} w_{xx}+w_{yy}+aw_{x}+bw_{y}+cw=0, \end{array}$$

where

(10) a=α1+iα2,  b=β1+iβ2,c=γ1+iγ2,  w=u+iv.

The Cotton invariants, corresponding to the complex elliptic equation (9), are (7) which split into the four invariants

(11) $$\begin{array}{c} \mu_{1}=\alpha_{1y}-\beta_{1x},\\ \mu_{2}=\alpha_{2y}-\beta_{2x},\\ H_{1}=\alpha_{1x}+\beta_{1y}+\frac{1}{2}\left(\alpha_{1}^{2}+\beta_{1}^{2}\right)-\frac{1}{2}\left(\alpha_{2}^{2}+\beta_{2}^{2}\right)-2\gamma_{1},\\ H_{2}=\alpha_{2x}+\beta_{2y}+\alpha_{1}\alpha_{2}+\beta_{1}\beta_{2}-2\gamma_{2}. \end{array}$$

These are precisely the Cotton-type invariants for the linear elliptic system (8). The simplest case is when the semiinvariants (11) are zero. In this case the elliptic PDE system (8) reduces to the Laplace system by linear transformation of the dependent variables. This is similar to the scalar linear elliptic PDE case.

Now for the system of elliptic equations (8), we derive the Cotton-type invariants by transforming the system of equations to the corresponding linear hyperbolic equations and then using the inverse transformations of the independent variables to convert the Laplace-type invariants to the Cotton-type invariants. By means of the transformations (3), the system of elliptic equations (8) can be mapped to the system of two linear hyperbolic type equations as follows:

(12) $$\begin{array}{c} u_{tz}+A_{1}u_{t}-A_{2}v_{t}+B_{1}u_{z}-B_{2}v_{z}+C_{1}u-C_{2}v=0,\\ v_{tz}+A_{2}u_{t}+A_{1}v_{t}+B_{2}u_{z}+B_{1}v_{z}+C_{2}u+C_{1}v=0, \end{array}$$

where

(13) $$\begin{array}{c} A_{1}=\frac{1}{4}\left(\alpha_{1}+i\beta_{1}\right),\;\;B_{1}=\frac{1}{4}\left(\alpha_{1}-i\beta_{1}\right),\;\;C_{1}=\frac{1}{4}\gamma_{1},\\ A_{2}=\frac{1}{4}\left(\alpha_{2}+i\beta_{2}\right),\;\;B_{2}=\frac{1}{4}\left(\alpha_{2}-i\beta_{2}\right),\;\;C_{2}=\frac{1}{4}\gamma_{2}. \end{array}$$

The system of hyperbolic equations (12) has four Laplace-type invariants [13]:

(14) $$\begin{array}{c} h_{1}=A_{1t}+A_{1}B_{1}-A_{2}A_{2}-C_{1},\\ h_{2}=A_{2t}+A_{1}B_{2}+A_{2}B_{1}-C_{2},\\ k_{1}=B_{1z}+A_{1}B_{1}-A_{2}B_{2}-C_{1},\\ k_{2}=B_{2z}+A_{1}B_{2}+A_{2}B_{1}-C_{2}. \end{array}$$

Now by the application of the transformations (3) and complex splits, the Laplace-type invariants (14) become the Cotton-type invariants (11). We therefore conclude the following result.

**Proposition 1**

For a class of a system of two linear elliptic equations (8) obtained from a complex base linear elliptic equation (9) or equivalent to a subsystem of two linear hyperbolic equations (12) by complex linear transformations of the independent variables (3), Cotton-type invariants either constructed by splitting of the complex Cotton invariants (7) of the complex base elliptic equation into real and imaginary parts or those computed by split of the Laplace-type invariants (14) of the system of linear hyperbolic equations are identical to (11).

## 3. Cotton-Type and Joint Invariants in General

In this section, Cotton-type and joint invariants for a general system of two linear elliptic equations are obtained. A general system of two linear elliptic equations is

(15) $$\begin{array}{c} u_{xx}+u_{yy}+a_{1}u_{x}+a_{2}v_{x}+b_{1}u_{y}+b_{2}v_{y}+c_{1}u+c_{2}v=0,\\ v_{xx}+v_{yy}+a_{3}u_{x}+a_{4}v_{x}+b_{3}u_{y}+b_{4}v_{y}+c_{3}u+c_{4}v=0. \end{array}$$

By means of the complex transformations of the independent variables (3), this system (15) is transformed into the system of two linear hyperbolic equations as follows:

(16) $$\begin{array}{c} u_{tz}+A_{1}u_{t}+A_{2}v_{t}+B_{1}u_{z}+B_{2}v_{z}+C_{1}u+C_{2}v=0,\\ v_{tz}+A_{3}u_{t}+A_{4}v_{t}+B_{3}u_{z}+B_{4}v_{z}+C_{3}u+C_{4}v=0, \end{array}$$

where

(17) $$\begin{array}{c} A_{1}=\frac{1}{4}\left(a_{1}+ib_{1}\right),\;\;B_{1}=\frac{1}{4}\left(a_{1}-ib_{1}\right),\;\;C_{1}=\frac{1}{4}c_{1},\\ A_{2}=\frac{1}{4}\left(a_{2}+ib_{2}\right),\;\;B_{2}=\frac{1}{4}\left(a_{2}-ib_{2}\right),\;\;C_{2}=\frac{1}{4}c_{2},\\ A_{3}=\frac{1}{4}\left(a_{3}+ia_{3}\right),\;\;B_{3}=\frac{1}{4}\left(a_{3}-ib_{3}\right),\;\;C_{3}=\frac{1}{4}c_{3},\\ A_{4}=\frac{1}{4}\left(a_{4}+ib_{4}\right),\;\;B_{4}=\frac{1}{4}\left(a_{4}-ib_{4}\right),\;\;C_{4}=\frac{1}{4}c_{4}. \end{array}$$

This system of linear hyperbolic equations (16) has five semi-invariants [12] under the linear change of dependent variables. They are [12]

(18) $$\begin{array}{c} I_{1}=k_{1}+k_{4},\;\;I_{2}=k_{5}+k_{8},\\ I_{3}=k_{1}k_{4}-k_{2}k_{3},\;\;I_{4}=k_{5}k_{8}-k_{6}k_{7},\\ I_{5}=k_{1}k_{5}+k_{2}k_{7}+k_{3}k_{6}+k_{4}k_{8}, \end{array}$$

where

(19) $$\begin{array}{c} k_{1}=A_{1}B_{1}+A_{3}B_{2}+A_{1t}-C_{1},\\ k_{2}=A_{1}B_{3}+A_{3}B_{4}+A_{3t}-C_{3},\\ k_{3}=A_{2}B_{1}+A_{4}B_{2}+A_{2t}-C_{2},\\ k_{4}=A_{2}B_{3}+A_{4}B_{4}+A_{4t}-C_{4},\\ k_{5}=A_{1}B_{1}+A_{2}B_{3}+B_{1z}-C_{1},\\ k_{6}=A_{3}B_{1}+A_{4}B_{3}+B_{3z}-C_{3},\\ k_{7}=A_{1}B_{2}+A_{2}B_{4}+B_{2z}-C_{2},\\ k_{8}=A_{3}B_{2}+A_{4}B_{4}+B_{4z}-C_{4}. \end{array}$$

The system of linear hyperbolic equations (16) also has the four joint invariants [12]

(20) $$\begin{array}{c} J_{1}=\frac{I_{2}}{I_{1}},\;\;J_{2}=\frac{I_{3}}{I_{1}^{2}},\;\;J_{3}=\frac{I_{4}}{I_{1}^{2}},\;\;J_{4}=\frac{I_{5}}{I_{1}^{2}}. \end{array}$$

We utilize the same approach as in the previous section. Indeed via the transformations (3), the Laplace-type invariants (18) transform to the five Cotton-type invariants

(21) H1=Im⁡(K1+K4)=Im⁡(K5+K8),H2=Re(K1+K4)=Re(K5+K8),H3=Im⁡(K1K4−K2K3)=Im⁡(K5K8−K6K7),H4=Re(K1K4−K2K3)=Re(K5K8−K6K7),H5=Re(K1K5+K2K7+K3K6+K4K8).

And the invariant equation is

(22) Im⁡(K1K5+K2K7+K3K6+K4K8)=0,

where

(23) K1=142(a12+b12+a2a3+b2b3+2a1x+2b1y−4c1) +i42(a2b3−a3b2−2a1y+2b1x),K2=142(a1a3+b1b3+a3a4+b3b4+2a3x+2b3y−4c3) +i42(a3b1−a1b3+a4b3−a3b4−2a3y+2b3x),K3=142(a1a2+b1b2+a2a4+b2b4+2a2x+2b2y−4c2) +i42(a1b2−a2b1+a2b4−a4b2−2a2y+2b2x),K4=142(a2a3+b2b3+a42+b42+2a4x+2b4y−4c4) +i42(a3b2−a2b3−2a4y+2b4x),K5=142(a12+b12+a2a3+b2b3+2a1x+2b1y−4c1) +i42(a3b2−a2b3+2a1y−2b1x),K6=142(a1a3+b1b3+a3a4+b3b4+2a3x+2b3y−4c3) +i42(a1b3−a3b1+a3b4−a4b3+2a3y−2b3x),K7=142(a1a2+b1b2+a2a4+b2b4+2a2x+2b2y−4c2) +i42(a2b1−a1b2+a4b2−a2b4+2a2y−2b2x),K8=142(a2a3+b2b3+a42+b42+2a4x+2b4y−4c4) +i42(a2b3−a3b2+2a4y−2b4x).

Note that we have an invariant equation here. This differs from the invariants of the split elliptic system of Section 2. We therefore have the following result.

**Proposition 2**

A general system of two linear elliptic equations (15) has the five Cotton-type invariants (21) and its coefficients satisfy the invariant condition (22).

Now the four joint invariants (20) reduce to the four invariants of the elliptic equations (15) and they are

(24) $$\begin{array}{c} \mu_{1}=\frac{\left(H_{1}^{2}-H_{2}^{2}\right)H_{3}+2H_{1}H_{2}H_{4}}{{\left(H_{1}^{2}-H_{2}^{2}\right)}^{2}+4H_{1}^{2}H_{2}^{2}},\\ \mu_{2}=\frac{\left(H_{1}^{2}-H_{2}^{2}\right)H_{4}-2H_{1}H_{2}H_{3}}{{\left(H_{1}^{2}-H_{2}^{2}\right)}^{2}+4H_{1}^{2}H_{2}^{2}},\\ \mu_{3}=\frac{\left(H_{1}^{2}-H_{2}^{2}\right)H_{5}}{{\left(H_{1}^{2}-H_{2}^{2}\right)}^{2}+4H_{1}^{2}H_{2}^{2}},\\ \mu_{4}=\frac{-2H_{1}H_{2}H_{5}}{{\left(H_{1}^{2}-H_{2}^{2}\right)}^{2}+4H_{1}^{2}H_{2}^{2}}, \end{array}$$

where the semi-invariants *H*_1_^2^ and *H*_2_^2^ are both not zero. The situation when both are zero occur for the Laplace system discussed earlier. We have thus obtained the Cotton-type and joint invariants for a general linear elliptic system of two equations (15) by using the Laplace-type and joint invariants of the general system of linear hyperbolic equations (16) by utilizing the known semi- and joint invariants of [12]. We thus state the following proposition.

**Proposition 3**

A general system of two linear elliptic equations (15) has the four joint invariants (24).

## 4. Applications

Here we present some examples for illustration. We consider *u*, *v*, $\bar{u}$, and $\bar{v}$ as dependent variables and *x*, *y*, *s*, and *t* as independent variables.

**Example 4**

Consider the system of two linear elliptic equations

(25) $$\begin{array}{c} u_{xx}+u_{yy}+\frac{2}{x}u_{x}+\frac{4}{y}u_{y}+\frac{2}{y^{2}}u=0,\\ v_{xx}+v_{yy}-\frac{4}{x}v_{x}-\frac{2}{y}v_{y}+2\left(\frac{1}{y^{2}}+\frac{3}{x^{2}}\right)v=0. \end{array}$$

This system transforms to the simplest elliptic equations

(26) $$\begin{array}{c} {\bar{u}}_{xx}+{\bar{u}}_{yy}=0,\;\;{\bar{v}}_{xx}+{\bar{v}}_{yy}=0, \end{array}$$

under the transformation

(27) $$\begin{array}{c} \bar{u}=xy^{2}u,\;\;\bar{v}=\frac{v}{x^{2}y}. \end{array}$$

The systems of elliptic equations (25) and (26) are transformable into each other as these systems have the same Cotton-type semi-invariants

(28) $$\begin{array}{c} H_{1}=H_{2}=H_{3}=H_{4}=H_{5}=0. \end{array}$$

**Example 5**

The system of elliptic equations

(29) uxx+uyy+(1−2y)ux+(1−2x)uy  +(x2+y2−x−y)u=0,vxx+vyy+(1−2y)vx+(1−2x)vy  +(x2+y2−x−y)v=0,

with the Cotton-type invariants

(30) $$\begin{array}{c} H_{1}=0,\;\;H_{2}=\frac{1}{4},\\ H_{3}=0,\;\;H_{4}=\frac{1}{64},\;\;H_{5}=\frac{1}{32}, \end{array}$$

reduces to the simple system of elliptic equations

(31) $$\begin{array}{c} {\bar{u}}_{xx}+{\bar{u}}_{yy}+{\bar{u}}_{x}+{\bar{u}}_{y}=0,\\ {\bar{v}}_{xx}+{\bar{v}}_{yy}+{\bar{v}}_{x}+{\bar{v}}_{y}=0, \end{array}$$

by the application of the transformation

(32) $$\begin{array}{c} \bar{u}=\exp\left(-xy\right)u,\;\;\bar{v}=\exp\left(-xy\right)v. \end{array}$$

The system (31) also has the Cotton-type invariants (30).

**Example 6**

The uncoupled system two of elliptic equations

(33) uxx+uyy+(2x+1)ux+(1−4y)uy  +(1x+6y2−2y)u=0,vxx+vyy+(1−2x)vx+(1+4y)vy  +(2y2+2y−1x+2x2)v=0,

has the Cotton-type invariants

(34) $$\begin{array}{c} H_{1}=0,\;\;H_{2}=\frac{1}{4},\\ H_{3}=0,\;\;H_{4}=\frac{1}{64},\;\;H_{5}=\frac{1}{32}. \end{array}$$

Therefore it is reducible to the simple system

(35) $$\begin{array}{c} {\bar{u}}_{xx}+{\bar{u}}_{yy}+{\bar{u}}_{x}+{\bar{u}}_{y}=0,\\ {\bar{v}}_{xx}+{\bar{v}}_{yy}+{\bar{v}}_{x}+{\bar{v}}_{y}=0, \end{array}$$

by means of the transformation

(36) $$\begin{array}{c} \bar{u}=\frac{x}{y^{2}}u,\;\;\bar{v}=\frac{y^{2}}{x}v. \end{array}$$

**Example 7**

Consider now the linear system of elliptic equations

(37) uxx+uyy+(2x+12)ux+(12−2y)uy  +12(4y2−1y+1x)u=0,vxx+vyy+(2x+12)vx+(12−2y)vy  +12(4y2−1y+1x)v=0,

which has the joint invariants

(38) $$\begin{array}{c} \mu_{1}=0,\;\;\mu_{2}=-\frac{1}{4},\;\;\mu_{3}=-\frac{1}{2},\;\;\mu_{4}=0. \end{array}$$

By using the transformation

(39) $$\begin{array}{c} s=\frac{x}{2},\;\;t=\frac{y}{2},\;\;\bar{u}=\frac{x}{y}u,\;\;\bar{v}=\frac{x}{y}v, \end{array}$$

the above system reduces to the simple system

(40) $$\begin{array}{c} {\bar{u}}_{ss}+{\bar{u}}_{tt}+{\bar{u}}_{s}+{\bar{u}}_{t}=0,\\ {\bar{v}}_{ss}+{\bar{v}}_{tt}+{\bar{v}}_{s}+{\bar{v}}_{t}=0, \end{array}$$

because this system has the joint invariant identical to the system (37).

**Example 8**

Finally, the coupled system of elliptic equations

(41) uxx+uyy+(2+1x)ux+2y3x−3/2vx−2yuy  +(1x−14x2+2y2)u−2y3x−5/2v=0,vxx+vyy+2x3/2y3ux+2(1−1x)vx+4yvy  +x1/2y3u+2(1y2−1x+1x2)v=0,

with the joint invariants

(42) $$\begin{array}{c} \mu_{1}=0,\;\;\mu_{2}=0,\;\;\mu_{3}=-1,\;\;\mu_{4}=0, \end{array}$$

simplifies to the system

(43) $$\begin{array}{c} {\bar{u}}_{ss}+{\bar{u}}_{tt}+{\bar{u}}_{s}+{\bar{u}}_{t}+{\bar{v}}_{s}+{\bar{v}}_{t}=0,\\ {\bar{v}}_{ss}+{\bar{v}}_{tt}+{\bar{u}}_{s}+{\bar{u}}_{t}+{\bar{v}}_{s}+{\bar{v}}_{t}=0, \end{array}$$

which has the same joint invariants as the system (41). The transformation that does this reduction is

(44) s=x+y,  t=x−y,u¯=xyu,  v¯=y2xv.

## 5. Conclusion

In this paper, we have derived the Cotton-type invariants for a special class of a system of two linear elliptic equations in two independent variables which arises from the complex split of a base complex linear elliptic equation. Moreover, the Cotton-type and joint invariants for a general system of two linear elliptic equations are also obtained. Laplace 1773, in his fundamental memoir, discussed two semi-invariants under the change of dependent variables of the scalar linear hyperbolic equation, known as Laplace invariants. Later, Cotton 1900 derived semi-invariants for the scalar linear elliptic equation, known as Cotton invariants. Linear hyperbolic and elliptic equations can be transformed into each other by the application of linear complex transformation of the independent variables. So do Laplace and Cotton invariants.

By a complex split, a complex scalar linear elliptic equation has been transformed into a system of two linear elliptic equations, which is a subclass of the general system of two linear elliptic equations. Cotton-type semi-invariants for this system of elliptic equations are obtained by two approaches. One is by splitting of the complex Cotton invariants that correspond to the complex base scalar linear elliptic equation into real and imaginary parts and the second by transformation of the subsystem of the linear elliptic equations into linear hyperbolic equations and application of the linear inverse transformations on the Laplace-type semi-invariants of the hyperbolic equations to deduce the Cotton-type invariants for the required subsystem of linear elliptic equations. It is found that the Cotton-type invariants by both approaches are the same. For a general system of linear elliptic equations, the Cotton-type and joint invariants have been constructed by transformation of the system of two linear elliptic equations into a system of two linear hyperbolic equations and thereafter applying the linear inverse transformations on the Laplace-type and joint invariants of [12] to deduce the Cotton-type and joint invariants for the linear system of elliptic equations.
